# Biomarker-Based Prediction of Treatment Outcomes in Pediatric Intussusception: A Retrospective Cohort Study

**DOI:** 10.3390/diagnostics16152431

**Published:** 2026-08-01

**Authors:** Karla Pehar, Danijela Jurić, Kristina Jurković, Marko Bašković

**Affiliations:** 1 School of Medicine, Catholic University of Croatia, Ilica 242, 10000 Zagreb, Croatia; 2 Department of Pediatric Surgery, Children’s Hospital Zagreb, Ulica Vjekoslava Klaića 16, 10000 Zagreb, Croatia; 3 School of Medicine, University of Zagreb, Šalata 3, 10000 Zagreb, Croatia; 4 Scientific Centre of Excellence for Reproductive and Regenerative Medicine, School of Medicine, University of Zagreb, Šalata 3, 10000 Zagreb, Croatia; 5 Croatian Academy of Medical Sciences, Kaptol 15, 10000 Zagreb, Croatia

**Keywords:** intussusception, intestinal obstruction, biomarkers, prognostic, predictive, spontaneous reduction, hydrostatic reduction, surgical management, children, pediatric surgery

## Abstract

**Background/Objectives**: Intussusception is a common pediatric emergency characterized by bowel telescoping, often leading to ischemia, necrosis, and perforation if untreated. Early identification of prognostic biomarkers could improve management strategies. This study aimed to evaluate the association between admission biomarkers and treatment outcomes in children with intussusception, to identify potential predictors. **Methods**: A retrospective analysis was conducted on 141 pediatric patients diagnosed with intussusception over ten years. Patients were stratified into three groups based on treatment outcome: spontaneous resolution, hydrostatic reduction, or surgery. Data collected included demographic, clinical, radiological, and laboratory parameters. **Results**: Among the cohort, 34% experienced spontaneous resolution, 27.7% underwent hydrostatic reduction, and 38.3% required surgery. Biomarkers such as erythrocyte count, hemoglobin, hematocrit, and platelets differed significantly across groups. Multivariate analysis identified erythrocyte count, platelet count, and potassium as variables independently associated with surgical management. Lower erythrocyte counts, higher platelet counts, and lower potassium were associated with increased likelihood of surgical intervention. The erythrocyte count, platelet count, and serum potassium showed ROC AUCs of 0.64, 0.65, and 0.70, respectively. **Conclusions**: Admission erythrocyte count, platelet count, and serum potassium were independently associated with the need for surgery but each showed only modest standalone discrimination. They are therefore best regarded as candidate variables for multivariable or composite predictive models rather than as standalone, clinically actionable triage markers. Further prospective studies are warranted to validate their incremental value within such models.

## 1. Introduction

Intussusception, characterized by the telescoping of a segment of the intestine into an adjacent distal segment, is recognized as one of the most common pediatric abdominal emergencies, predominantly affecting children under two years of age [1]. This condition results from the invagination of the proximal bowel (intussusceptum) into the distal bowel (intussuscipiens), leading to vascular congestion, edema, and, if untreated, ischemia, necrosis, perforation, and peritonitis. Although the etiology is idiopathic in the majority of cases, approximately 25% are associated with identifiable pathological lead points, such as Meckel’s diverticulum, polyps, duplication cysts, or neoplasms. The clinical presentation varies, but classic symptoms include sudden onset of colicky abdominal pain, vomiting, palpable abdominal mass, and in some cases, currant-jelly stools indicative of mucosal hemorrhage [2,3,4].

Epidemiologically, intussusception predominantly affects infants and toddlers, with the highest incidence occurring between four and thirty-six months of age [5,6]. It accounts for roughly 30% of bowel obstructions in this age group, making it a significant pediatric emergency worldwide. While most cases are idiopathic and occur in otherwise healthy children, age-specific differences exist. The condition exhibits a slight male predominance with a male-to-female ratio of approximately 3:2, and predominantly occurs in children who are well-nourished and otherwise healthy [6,7,8].

The pathogenesis of intussusception involves the abnormal peristaltic activity dragging a focal lesion or hypertrophied lymphoid tissue, such as enlarged Peyer’s patches, into the distal bowel, thus functioning as a lead point [1,9]. Viral infections, notably rotavirus and adenoviruses, have been implicated as potential triggers, stimulating lymphoid hyperplasia and increasing the risk of intussusception [10,11]. Seasonal variations and associations with certain vaccines further support the role of infectious etiologies [12]. Other predisposing factors include bacterial enteritis, parasitic infections, and underlying disorders such as cystic fibrosis or immunological conditions. In some cases, structural anomalies like Meckel’s diverticulum or neoplasms serve as focal lead points that facilitate invagination [1,7,13].

Clinically, the presentation of pediatric intussusception can be both typical and atypical [14]. The classic triad involves sudden, intermittent, severe abdominal pain, palpable sausage-shaped abdominal mass, and rectal bleeding—sometimes manifesting as “currant-jelly” stool. However, this triad is present in fewer than 15% of cases, and children may exhibit nonspecific symptoms such as lethargy, altered consciousness, or episodic pain without overt bleeding [1,15,16]. In infants, symptoms can be subtle or atypical, often leading to misdiagnosis or delayed treatment [17]. The severity of presentation correlates with the duration and extent of bowel ischemia, emphasizing the importance of early recognition [18].

Diagnostic evaluation relies heavily on imaging modalities, primarily ultrasonography, which offers high sensitivity and specificity in experienced hands. The characteristic “target sign” on ultrasound is diagnostic of intussusception, and it allows for real-time monitoring of reduction efforts [19]. Plain abdominal radiographs are primarily used to exclude perforation or other causes of obstruction but lack diagnostic specificity. Computed tomography (CT) can identify intussusception and underlying lead points but is generally reserved for atypical or complicated cases due to radiation concerns [20,21]. Laboratory tests may reveal markers of inflammation or ischemia, such as elevated white blood cell counts, C-reactive protein, lactate, or D-dimer, which can suggest severity and guide management [22,23].

The management of pediatric intussusception depends on the clinical presentation, stability of the patient, and imaging findings. Non-operative reduction using hydrostatic or pneumatic enemas under ultrasound or fluoroscopic guidance remains the first-line treatment in stable children without signs of perforation or peritonitis, boasting success rates between 70% and 85% [24,25]. When successful, this approach is both diagnostic and therapeutic, avoiding the need for surgery. However, in cases of perforation, shock, or failed non-operative attempts, surgical intervention becomes necessary. Surgery may involve manual reduction or bowel resection if necrosis or a pathological lead point is identified [26]. Minimally invasive laparoscopic techniques are increasingly favored due to favorable recovery profiles and diagnostic accuracy [27].

In recent years, research has increasingly focused on identifying predictive and prognostic factors that can facilitate early diagnosis, assess severity, and forecast treatment outcomes in children with intussusception. Traditional clinical signs and imaging findings are complemented by emerging biomarkers, including hematological indices, inflammatory cytokines, and molecular markers, which hold promise for improving risk stratification [28]. In summary, while the clinical and radiological features of pediatric intussusception are well-characterized, variability in presentation and progression underscores the necessity for reliable predictive and prognostic markers. These factors could enhance early diagnosis, guide management decisions, and improve patient outcomes by enabling tailored, timely interventions. The integration of traditional clinical assessment with emerging biomarkers and advanced imaging techniques is poised to refine the current approach to this common yet potentially severe pediatric condition.

The main objective of the study was to investigate the association between levels of selected biomarkers, measured at admission, and treatment outcomes in pediatric patients with intussusception, in order to identify potential indicators of treatment success and clinical prognosis.

## 2. Materials and Methods

### 2.1. Patients

**Figure 1:**
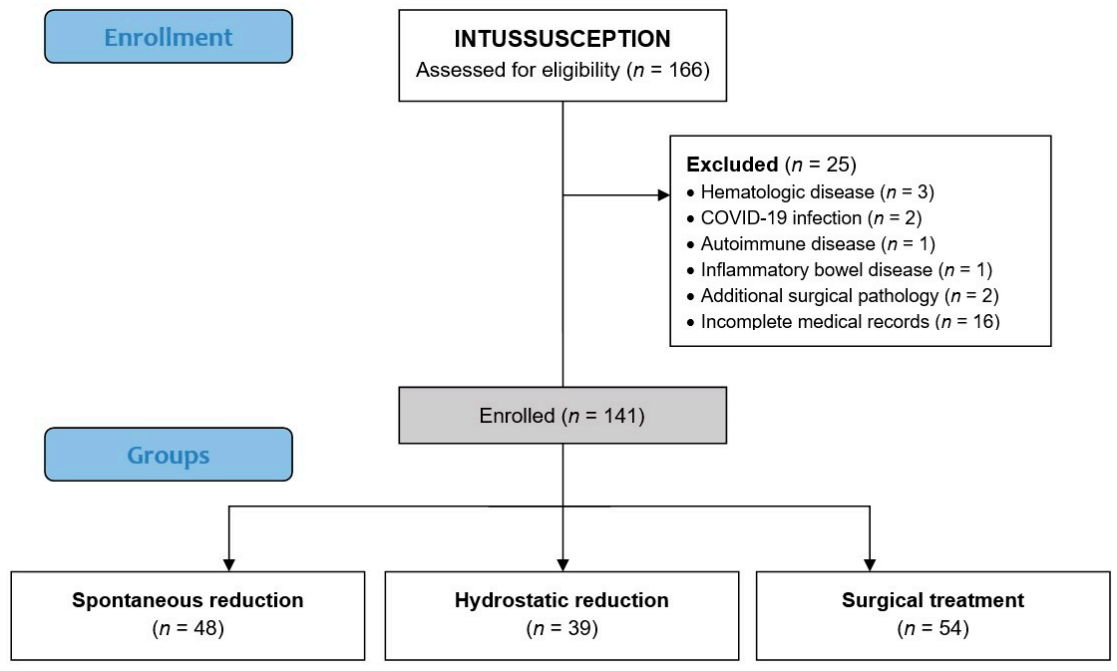
Flowchart of the study.

A retrospective review of electronic patient records was conducted using the hospital information system (IN2 BIS^®^, *v216.0.000*, Constellation Software Inc., Toronto, ON, Canada) to identify eligible participants for this study. The search included all patients treated at the Children’s Hospital Zagreb with a diagnosis of intussusception (International Classification of Diseases, 10th Revision [ICD-10], code K56.1) over a ten-year period from 1 January 2016 to 1 January 2026. Inclusion criteria were all patients in whom the diagnosis of intussusception was confirmed by ultrasound, following which they were admitted for observation and treatment. Exclusion criteria included patients with hematologic disease, COVID-19 infection, autoimmune disease, inflammatory bowel disease, those found to have additional surgical pathology intraoperatively, and patients with incomplete medical records. Following the identification of eligible patients, the cohort was stratified into three groups based on treatment outcome: (1) patients with spontaneous resolution of intussusception, (2) patients who underwent hydrostatic reduction, and (3) patients requiring surgical intervention. The study flow diagram is shown in Figure 1.

### 2.2. Study Design

The following parameters were recorded for each patient: gender, age, season of admission, comorbidities, presence of confirmed infection at the time of admission, type of infection, use of antibiotics at the time of admission, duration of symptoms, history of an intermittent pattern of pain, visual analog scale for children older than 8 years, stool consistency at the time of admission, presence of bloody stool, presence of nausea or vomiting at the time of admission, body temperature at the time of admission, presence of palpable abdominal tenderness at the time of admission, and location of the mentioned tenderness. The ultrasound parameters recorded were the type and number of intussusceptions, location(s) of intussusception(s), size of intussusception, pathological lead point, signs of ischemia or reduced blood flow to the bowel wall, bowel wall edema, presence of free fluid in the abdomen, and recurrence of intussusception within 48 h of the first ultrasound performed at admission. For surgically treated patients, additional data were recorded on attempted hydrostatic reduction, type of surgical procedure, duration of surgical procedure, intraoperatively determined type and location of intussusception, intraoperatively determined lead point, intraoperatively determined size of intussusception, presence of bowel perforation, size of resected bowel, perioperative and postoperative antibiotic administration, and type of antibiotic administered. Total length of stay (LOS) was also recorded. The following parameters (biomarkers) of interest were determined from the blood at the time of admission: erythrocytes, hemoglobin, hematocrit, mean corpuscular volume (MCV), mean corpuscular hemoglobin (MCH), mean corpuscular hemoglobin concentration (MCHC), red cell distribution width–coefficient of variation (RDW-CV), platelets, mean platelet volume (MPV), leukocytes, neutrophils (percentage, absolute), lymphocytes (percentage, absolute), monocytes (percentage, absolute), eosinophils (percentage, absolute), basophils (percentage, absolute), immature granulocytes (IG) (percentage, absolute), C-reactive protein (CRP), pH, partial pressure of carbon dioxide (pCO_2_), partial pressure of oxygen (pO_2_), bicarbonate (HCO_3_), total CO_2_, base excess (BE), oxygen saturation (sO_2_), potassium, sodium, chlorides, calcium, glucose, lactates, oxyhemoglobin, carboxyhemoglobin, methemoglobin, and deoxyhemoglobin. Based on certain previous parameters, NLR (Neutrophil-to-Lymphocyte Ratio), NMR (Neutrophil-to-Monocyte Ratio), MLR (Monocyte-to-Lymphocyte Ratio), PLR (Platelet-to-Lymphocyte Ratio), SII (Systemic Immune-Inflammation Index: platelet count × neutrophil count/lymphocyte count), and SIRI (Systemic Inflammatory Response Index: neutrophil count × monocyte count/lymphocyte count) were calculated.

### 2.3. Diagnostic Procedures

Capillary or venous blood samples were collected by trained nursing staff or laboratory technicians following standard procedures. The blood samples were analyzed using automated hematology analyzers, specifically the XN-1000™ (Sysmex Corporation, Kobe, Japan) or the XS-800i™ (Sysmex Corporation, Kobe, Japan). C-reactive protein (CRP) levels were measured from capillary or venous blood samples using the D7-CRP Hematology Analyzer (Dymind Biotechnology, Shenzhen, China), or on AU400 or AU680 Clinical Chemistry Analyzer (Beckman Coulter, Brea, CA, USA). Blood gas analysis was conducted using the RAPIDPoint^®^ 500 System (Siemens Healthcare GmbH, Erlangen, Germany). All ultrasonographic examinations were performed and interpreted by consultant pediatric radiologists; over the study period the examinations were reported by six consultants, each with more than ten years of experience, with a single radiologist reporting each case. Examinations were performed using a Logiq E9^®^ system (GE Healthcare Systems, Chicago, IL, USA) according to the institution’s standard imaging protocol. The diagnosis of intussusception was established using predefined criteria—the “target” (or “doughnut”) sign in the transverse plane and the “pseudokidney” sign in the longitudinal plane—and each examination documented the type, number, and location of the intussusception(s), its size, the presence of a pathological lead point, bowel-wall vascularity assessed with color Doppler (signs of ischemia/reduced perfusion), bowel-wall edema, and free intraperitoneal fluid. The same protocol defined the indications and contraindications for hydrostatic reduction, and the decision to attempt hydrostatic reduction was made by the reporting radiologist. The admission ultrasound was typically the indication for admission and was generally performed within one hour of arrival; venous or capillary blood for the biomarker panel was likewise drawn within approximately one hour of admission. Because ultrasound was generally performed and interpreted before the laboratory results became available, the reporting radiologists were typically unaware of the biomarker values at the time of interpretation. When indicated, hydrostatic reduction followed the diagnostic ultrasound, whereas the indication for surgery was set by the attending pediatric surgeon. In STARD terms, admission ultrasound served as the index test for the diagnosis and anatomical classification of intussusception, while intraoperative findings served as the reference standard for the anatomical type and pathological lead point and were therefore available only for surgically treated patients; in the remaining patients the diagnosis was supported by the ultrasound findings together with the clinical course (spontaneous resolution or successful hydrostatic reduction). When the ultrasound classification differed from the intraoperative findings, the intraoperative findings were taken as definitive; such discordance did not alter management, as the indication for surgery was set by the attending surgeon on clinical grounds rather than by the radiological classification alone.

### 2.4. Hydrostatic Reduction

Hydrostatic reduction in ileocolic intussusception at our institution is performed by experienced radiologists under continuous ultrasound guidance using a saline enema to create a controlled retrograde pressure. The procedure involves placing a saline container approximately one meter above the patient to establish a consistent hydrostatic pressure gradient. In keeping with widely adopted enema-reduction protocols (the “rule of threes”), the saline reservoir is not raised above approximately 1–1.2 m above the patient, corresponding to a maximum hydrostatic column pressure of about 100–120 cm H_2_O, in order to minimize the risk of perforation. The saline is instilled into the rectum through a lubricated catheter, with real-time ultrasound monitoring to monitor the progress of the reduction. Successful reduction is indicated by the disappearance of the intussusception and the appearance of water and bubbles in the terminal ileum. The radiologist may increase the hydrostatic pressure by elevating the saline container if initial attempts are unsuccessful, taking into account the patient’s clinical condition. A maximum of three reduction attempts is permitted in a single session, each lasting up to approximately three minutes, with the child’s clinical and hemodynamic stability reassessed between attempts. Reduction is considered to have failed when the intussusception cannot be reduced after three attempts, in which case the child is referred for surgical management. Ultrasound is used throughout the procedure to ensure safety and effectiveness, and the procedure continues until the intussusception resolves or signs of complications appear. Ultrasound is used after the procedure to confirm the success of the reduction and to assess any possible side effects and complications.

### 2.5. Surgical Procedures

Surgical intervention in our institution is indicated in cases of failed non-operative reduction, signs of intestinal ischemia, gangrene, or perforation, radiologically proven pathological lead point, patient instability or peritonitis, and contraindications for non-operative methods. At our institution, surgical management of pediatric intussusception is performed through an approach involving laparotomy under general anesthesia. The procedure begins with a midline laparotomy (*laparotomia mediana explorativa*) to gain access to the abdominal cavity, allowing thorough exploration and assessment of the bowel. Manual reduction in the intussuscepted segments (*desinvaginatio manualis*) is attempted to restore normal bowel anatomy. If manual reduction is successful, further examination of the bowel is conducted to identify any pathological lead points, such as Meckel’s diverticulum, tumors, or hypertrophic lymphoid tissue. In cases where reduction is difficult or a lead point is suspected, resection of the affected bowel segment is performed, followed by appropriate anastomoses. Additional procedures may include appendectomy, especially if the appendix appears inflamed or as a prophylactic measure, and lymph node extirpation when enlarged lymph nodes are encountered. Before closure, abdominal lavage is routinely carried out to minimize the risk of postoperative infection. The choice of resection versus conservative reduction depends on intraoperative findings, bowel viability, and the presence of pathological lead points, aligning with current surgical standards for effective management of pediatric intussusception.

### 2.6. Outcome Measures

The primary outcome of this study is to evaluate the association between levels of selected biomarkers—measured at admission—and the treatment outcome in pediatric patients with intussusception. Specifically, the primary outcome is the ability of these biomarkers to predict whether a patient will experience spontaneous resolution, respond successfully to hydrostatic reduction, or require surgical intervention. This assessment aims to identify potential biomarkers that can serve as early indicators of treatment success or failure, thereby facilitating tailored management strategies. Secondary outcomes of the study are to compare patient groups by outcome in terms of their demographic, clinical, and radiological characteristics at admission.

### 2.7. Statistical Analysis

Continuous variables were summarized as mean ± SD when normally distributed (Shapiro–Wilk test) and as median (IQR) otherwise; categorical variables were summarized as frequencies. Categorical variables were compared with Fisher’s exact or chi-square tests; continuous variables with Student’s *t*-test or the Mann–Whitney U test for two groups, and one-way ANOVA or the Kruskal–Wallis test for three groups, as appropriate to the distribution. Correlations used Pearson’s or Spearman’s coefficient. Independent predictors of surgical treatment were identified by multivariable binary logistic regression, with odds ratios expressed per one-standard-deviation increase, and diagnostic performance was assessed by receiver operating characteristic (ROC) analysis. Analyses were performed in XLSTAT^®^ (v2025.1, Addinsoft, Paris, France) within Microsoft Excel^®^, with significance set at *p* < 0.05. Because a large biomarker panel was examined, analyses were structured a priori into a primary tier—the erythrocyte and platelet counts, biologically plausible predictors of surgery carried into the multivariable and ROC models, and an exploratory tier of all other biomarkers. Exploratory three-group comparisons were corrected for the false discovery rate (Benjamini–Hochberg) across the 46-variable panel (adjusted q < 0.05) and are reported as hypothesis-generating. Effect sizes accompany *p*-values: η^2^ with a bootstrap 95% CI (2000 resamples) for group comparisons, and odds ratios with 95% CIs for the multivariable models. Agreement between the ultrasound-based and intraoperative anatomical classification of intussusception was quantified with Cohen’s κ, together with the percentage agreement and a cross-tabulation, in the subgroup of surgically treated patients for whom the intraoperative reference standard was available. The study was reported in accordance with the applicable items of the STARD 2015 statement.

### 2.8. Sensitivity Analysis—Restricted Cohort

To reduce confounding by anatomical type and group heterogeneity, a sensitivity analysis was restricted to children with ileocolic or ileocecocolic intussusception (*n* = 82), the types for which non-operative reduction and surgery are the principal options, dichotomized into non-operative (spontaneous resolution or hydrostatic reduction; *n* = 41) and surgical (*n* = 41) groups. Continuous variables were compared with the Mann–Whitney U test and categorical variables with Fisher’s exact test. A parsimonious multivariable binary logistic regression model (surgical treatment as outcome; erythrocyte count, platelet count, age, and symptom duration as covariates) tested whether the candidate biomarkers predicted surgery independently, with odds ratios reported per 1-SD increase. Discriminatory performance of erythrocyte count, platelet count, and serum potassium was assessed by ROC analysis, reporting the AUC, the Youden-optimal cut-off, and the corresponding sensitivity, specificity, and predictive values.

## 3. Results

During the observed ten-year period, a total of 166 children with a diagnosis of intussusception were hospitalized at the Children’s Hospital Zagreb. After applying the exclusion criteria, 141 patients remained for analysis. In 48 (34%) patients, spontaneous reduction occurred, in 39 (27.7%), hydrostatic reduction was performed, and in 54 (38.3%), surgical treatment was indicated (Figure 1).

The median age of all patients was 2.2 years (IQR 1.3–4.4). The youngest patient was 2 months old, and the oldest was 15.8 years old. There were 84 (59.6%) boys and 57 (40.4%) girls. Comorbidities that were not exclusion criteria were recorded in 11 (7.8%) patients. At the time of admission, 32 (22.7%) patients had verified some form of infection (most often adenovirus), while 12 (8.5%) patients had some antibiotic therapy. The largest number of patients, 43 (30.5%), were admitted during the autumn, and the largest number of admissions, 15 (10.6%), were recorded around 12 o’clock in the afternoon. The duration of abdominal pain before admission was classified according to established thresholds: 58 (41.1%) patients presented within 24 h, 33 (23.4%) at 24–48 h, and 50 (35.5%) after 48 h. The distribution did not differ significantly across treatment groups. Patients or their parents reported an intermittent pattern of pain in 77 (54.6%) patients. In patients in whom pain intensity could be assessed using a visual analog scale (0–10), the mean value was 2.6 (min—1, max—7). Nausea or vomiting was present in 93 (66%) patients on admission. In the majority, 94 (66.7%) patients, stool was normal, while blood in the stool was present in 25 (17.7%) patients. On admission, the majority of patients were afebrile; only seven (5%) were febrile (≥38.0 °C), with no significant difference across treatment groups. Palpatory abdominal tenderness on admission was present in 53 (37.6%) patients, mostly of a diffuse nature. Demographic and clinical data at the time of admission of patients between groups are shown in Table 1.

Ultrasound examination in the entire cohort revealed 78 (55.4%) ileocolic intussusceptions, four (2.8%) ileocecocolic intussusceptions, 53 (37.6%) ileoileal intussusceptions, and three (2.1%) jejunojejunal intussusceptions, while in two (1.4%) patients both ileocolic and ileoileal intussusception were detected simultaneously, and in one (0.7%) both jejunojejunal and ileoileal intussusception were detected simultaneously. A lead point was suspected based on ultrasound in six cases, all of which were ultimately treated surgically. The mean size of the intussusception on ultrasound was 4.2 ± 3.5 cm. Ultrasound revealeintestinal wall edema in 43 (30.5%) patients, and signs of ischemia in seven (5%) patients. The presence of free fluid in the abdomen was determined in 54 (38.3%) patients. In groups 1 and 2, recurrence of intussusception within 48 h was recorded in three patients, one in group 1, and two in group 2. In group 3, hydrostatic reduction was attempted in 19 (35.2%) patients, which was unsuccessful. Patient radiological data between groups are shown in Table 2.

The mean values (±SD) of blood count variables at admission by group, along with the corresponding *p*-values, are shown in Table 3.

The mean values (±SD) of the ratios and indexes by group, along with the corresponding *p*-values, are shown in Table 4.

The mean values (±SD) of acid-base status variables at admission by group, along with the corresponding *p*-values, are shown in Table 5.

Based on previous analyses of blood parameters, erythrocyte count, hemoglobin, hematocrit, platelet count, lymphocyte count, and potassium measured at admission were evaluated as candidate predictors of surgical treatment in a multivariable binary logistic regression model (Table 6).

Surgical treatment was modeled by multivariable binary logistic regression. Because erythrocyte count, hemoglobin, and hematocrit were strongly collinear, hemoglobin and hematocrit were excluded and the erythrocyte count was retained to represent the red-cell indices. Lower erythrocyte counts (OR 0.58 per 1 − SD; 95% CI 0.34–0.99; *p* = 0.045), higher platelet counts (OR 1.97; 95% CI 1.07–3.64; *p* = 0.029), and lower potassium (OR 0.52; 95% CI 0.29–0.91; *p* = 0.021) were independently associated with the need for surgery, whereas the lymphocyte count was not (OR 0.91; 95% CI 0.50–1.67; *p* = 0.771).

**Figure 2:**
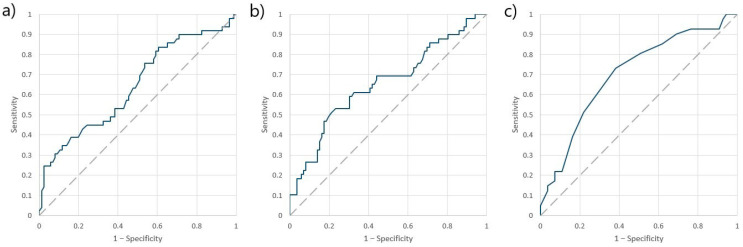
ROC curve for: (**a**) erythrocyte count, (**b**) platelet count, and (**c**) serum potassium.

ROC analysis demonstrated the diagnostic performance of erythrocyte count in predicting the need for surgical treatment, with an AUC of 0.64 (95% CI [0.5, 0.79]), standard error (SE) = 0.07, and *p* = 0.048. At a cutoff value of 3.98, erythrocyte count yielded a sensitivity of 24.49%, specificity of 97.67%, PPV of 85.71%, and NPV of 69.42%. ROC analysis demonstrated the diagnostic performance of platelet count in predicting the need for surgical treatment, with an AUC of 0.65 (95% CI [0.56, 0.75]), SE = 0.05, and *p* = 0.002. At a cutoff value of 377, platelet count yielded a sensitivity of 46.94%, specificity of 82.56%, PPV of 60.53%, and NPV of 73.20%. ROC analysis of serum potassium yielded an AUC of 0.70 (95% CI [0.60, 0.81]; SE = 0.06; *p* < 0.001); at a cutoff of 4.3 mmol/L, lower potassium gave a sensitivity of 73.17%, specificity of 61.82%, PPV of 58.82%, and NPV of 75.56%, with positive and negative likelihood ratios of 1.92 (1.31–2.81) and 0.43 (0.25–0.75). Likelihood ratios were modest; for erythrocyte count, the positive and negative likelihood ratios were 10.5 (95% CI 2.5–45.1) and 0.77 (0.66–0.91), with sensitivity 24.5% (14.6–38.1) and specificity 97.7% (91.9–99.4); for platelet count, they were 2.69 (1.56–4.65) and 0.64 (0.49–0.85), with sensitivity 46.9% (33.7–60.6) and specificity 82.6% (73.2–89.1) (Figure 2).

When the analysis was restricted to children with ileocolic or ileocecocolic intussusception (*n* = 82), 41 were managed non-operatively (three spontaneous resolutions and 38 hydrostatic reductions) and 41 required surgery. Within this anatomically homogeneous cohort, several clinical characteristics still differed between groups: children who required surgery were younger (median 14 vs. 22 months; *p* = 0.018), had a longer symptom duration (55 vs. 24 h; *p* = 0.006), and more often presented with bloody stool (41.5% vs. 7.3%; *p* < 0.001) and ultrasound bowel-wall edema (42.5% vs. 19.5%; *p* = 0.032), whereas sex, ultrasound signs of ischemia, free fluid, and suspected lead point did not differ significantly. The principal hematological findings of the full-cohort analysis were preserved. Erythrocyte count was lower (4.29 vs. 4.55 ×10^12^/L; *p* = 0.020) and platelet count higher (379 vs. 318 ×10^9^/L; *p* = 0.005) in the surgical group, with concordant differences in hemoglobin (*p* = 0.048) and hematocrit (*p* = 0.017). Eosinophil counts were lower and immature granulocytes higher in the surgical group, and the metabolic profile again distinguished it, with lower potassium (*p* < 0.001) and higher glucose (*p* < 0.001), whereas the leukocyte-derived inflammatory ratios did not differ significantly (Table S1).

In a multivariable logistic regression model adjusted for age and symptom duration, both erythrocyte count (OR 0.45; 95% CI 0.25–0.80; *p* = 0.007) and platelet count (OR 2.71; 95% CI 1.34–5.49; *p* = 0.006) remained independent predictors of surgery, whereas age (*p* = 0.087) and symptom duration (*p* = 0.382) did not. On ROC analysis, erythrocyte count predicted surgery with an AUC of 0.653 (95% CI 0.531–0.774; cut-off ≤ 4.26 ×10^12^/L; sensitivity 50.0%, specificity 78.0%) and platelet count with an AUC of 0.682 (95% CI 0.564–0.800; cut-off ≥ 365 ×10^9^/L; sensitivity 60.5%, specificity 82.9%), comparable to, and numerically slightly higher than, the full-cohort values. The corresponding likelihood ratios were also modest: positive and negative likelihood ratios of 2.28 (95% CI 1.18–4.40) and 0.64 (0.45–0.92) for erythrocyte count and 3.55 (1.72–7.30) and 0.48 (0.31–0.72) for platelet count; the 95% confidence intervals for sensitivity and specificity were 34.8–65.2% and 63.3–88.0% (erythrocytes) and 44.7–74.4% and 68.7–91.5% (platelets). Although serum potassium was not entered into this model, it showed even stronger discrimination in the restricted cohort, with an ROC AUC of 0.76 (95% CI 0.64–0.88; cut-off 4.1 mmol/L; sensitivity 58.1%, specificity 82.1%; positive and negative likelihood ratios of 3.25 and 0.51) (Table S2).

Among the 54 pediatric patients who underwent surgical management for intussusception, successful manual reduction was achieved in 52 cases. Of these, 36 patients did not require bowel resection. In 16 cases, bowel resection with primary end-to-end anastomosis was necessary. In two cases, manual reduction was not feasible intraoperatively. Of these, one patient required bowel resection with primary end-to-end anastomosis, while the other necessitated initial stoma formation with the subsequent formation of continuity. Intraoperatively, according to the type of intussusception, 36 were ileocolic, eight ileocecocolic, eight ileoileal, one jejunojejunal, and in one case, both ileocolic and ileoileal types were determined. Agreement between the ultrasound-based and the intraoperative anatomical classification was moderate (Cohen’s κ = 0.59, 95% CI 0.37–0.80; 43 of 54 cases, 79.6%). As for the lead points, they were determined intraoperatively in 12 cases, of which in six cases it was Meckel’s diverticulum, in four cases it was a lymph node, and in two cases it was a polyp. The mean length of the intraoperatively determined intussusception was 9.9 cm (min—2 cm, max—35 cm), and the mean length of the resected portion of the intestine was 14.7 cm (min—5 cm, max—31 cm), while visible intraoperative intestinal perforation was found in three patients. The mean duration of the operation was 76.6 ± 39.5 min (min—30 min, max—180 min). Perioperatively and postoperatively, antibiotics were prescribed in 33 patients, most often a combination of cefuroxime + metronidazole, and gentamicin + metronidazole.

The mean length of stay was 5.1 ± 3.3 days, for the spontaneous reduction group 2.9 ± 1.4 days, for the hydrostatic reduction group 3.2 ± 1.5 days, and for the surgically treated group 8.4 ± 2.8 days (*p* < 0.001, G_1_/G_3_, G_2_/G_3_).

## 4. Discussion

In this retrospective cohort of children with intussusception, admission hematological parameters—most notably erythrocyte and platelet counts—were the findings most closely associated with treatment outcome. Children who ultimately required surgery tended to present with lower red-cell indices and higher platelet counts than those whose intussusception resolved spontaneously or was reduced hydrostatically, and both remained independent predictors of the need for surgery on multivariate analysis, albeit with only moderate discriminatory performance. By contrast, the leukocyte-based inflammatory markers and derived inflammatory ratios emphasized in several earlier reports were not discriminating in our cohort, whereas the surgical group was distinguished among the biochemical parameters chiefly by metabolic and electrolyte disturbances. In particular, lower serum potassium was itself an independent predictor of surgery and showed the highest discrimination of any single marker. Taken together, these findings suggest that simple, routinely available blood-count parameters may aid early risk stratification, while underscoring that no single marker is sufficient in isolation.

Because the three treatment groups corresponded only loosely to distinct anatomical types, with the spontaneously resolving group consisting predominantly of transient ileoileal intussusception (87.5%) and the hydrostatically reduced group almost exclusively of ileocolic intussusception (97.4%), the associations between biomarkers and outcome could in principle have reflected differences in anatomical type, age, or clinical entity rather than a true relationship with disease severity. To examine this, we repeated the analysis in the anatomically homogeneous subset of children with ileocolic or ileocecocolic intussusception. The main findings were preserved: lower erythrocyte and higher platelet counts remained associated with the need for surgery and, critically, both retained independent predictive value after adjustment for age and symptom duration—the clinical factors that continued to differ between the non-operative and surgical groups within this subset. The discriminatory performance of both markers was at least as good as in the full cohort. These results indicate that the association between admission red-cell and platelet indices and the need for surgery is not merely an artifact of pooling distinct anatomical entities, and they strengthen the case for these routinely available parameters as early markers of severity in classic ileocolic intussusception. Nevertheless, the restricted cohort was small (82 children, 41 surgical events), the optimal cut-offs should be considered hypothesis-generating, and prospective validation in ileocolic intussusception specifically remains warranted.

Consistent with previous reports, our cohort showed a slight male predominance, and almost a quarter of children developed intussusception following a viral infection [7,29]. The median symptom duration was 24 h, longer than the 12 h reported by Acer-Demir et al., although, as in that study, vomiting was the leading symptom, present in two-thirds of patients [18]. An intermittent pattern of pain was recorded in about half, in keeping with Guo et al. [17]. Bloody stool was more frequent in our series (almost 18%) than in the case series of Li et al. (6%) [14]. Finally, pathological ultrasound signs at admission, bowel wall edema, signs of ischemia, and free fluid were, as in other studies, more prevalent among children who ultimately required surgery than among those with spontaneous or hydrostatic reduction [30,31].

When the duration of abdominal pain at presentation was stratified according to established thresholds, the proportion of children presenting after more than 48 h was highest in the surgical group (48.1%), compared with 31.3% in the spontaneous-resolution group and 23.1% in the hydrostatic-reduction group; however, this difference did not reach statistical significance (*p* = 0.101). This directional trend is nonetheless consistent with the wider literature, in which a longer symptom duration is a recognized predictor of failed non-operative reduction and the eventual need for surgery. Fike et al. found that a symptom duration exceeding 24 h was significantly more frequent among children with failed enema reduction [32], and a systematic review and meta-analysis of 38 studies comprising 40,133 children reported that a symptom duration shorter than 24 h was associated with successful reduction (OR 3.81; 95% CI 2.15–6.76). The absence of a statistically significant association in our cohort may reflect its comparatively modest sample size and the retrospective ascertainment of symptom onset. Similarly, only a small proportion of our patients were febrile on admission (5%), and the afebrile/febrile distribution did not differ significantly across treatment groups (*p* = 0.231). This contrasts with the meta-analysis by Kim et al., in which fever was significantly associated with failed enema reduction [33], a discrepancy most plausibly explained by the low number of febrile children in our cohort and the consequently limited statistical power.

Although elevated white blood cell counts (>10 × 10^9^/L and >20 × 10^9^/L), neutrophilia (>9420/cc), and higher NLR values (>1.2, >4.52, or >5.72) have frequently been linked to the need for surgery, ischemia, bowel necrosis, or a worse prognosis, we found no such associations, suggesting that these parameters lack sufficient predictive power when considered in isolation [34,35,36,37,38,39,40,41,42,43,44]. Likewise, and in contrast to Tuşat et al. [36] and Chen et al. [45], PLR did not differ significantly between children managed non-operatively and those requiring surgery. We did, however, find significantly higher absolute platelet counts in the surgical group, with multivariate analysis confirming higher platelet levels as a predictor of surgical treatment (OR 1.97 per 1 − SD; 95% CI 1.07–3.64; *p* = 0.029), consistent with Liu et al. [41]. As acute-phase reactants that rise with inflammation and tissue injury and mediate both inflammation and thrombosis, elevated platelets may reflect a more intense systemic inflammatory response and compromised bowel viability, including ischemia or necrosis, that ultimately necessitates surgery [46,47,48].

In our cohort, mean CRP was highest in the surgical group but the difference was not statistically significant, indicating limited prognostic value here. This contrasts with several studies linking elevated CRP to surgical need, bowel resection, and intestinal necrosis, with reported cut-offs ranging from approximately 3.4 mg/dL to over 11 mg/dL at adequate sensitivity and specificity—a consistent association that supports CRP’s role in risk stratification [22,37,38,39,40,41,45,49,50,51,52,53,54]. A range of other inflammatory and immune markers has likewise been implicated: IL-6 above 1.6 pg/mL and higher concentrations of cytokines such as IL-2, IL-4, IL-10, and TNF-α correlate with greater disease activity and tissue damage [50,55,56], serum neopterin is elevated in complicated cases, reflecting macrophage activation [56], MCP-1 is associated with early relapse and elevated endotoxins with bacterial translocation and mucosal compromise [57], and altered levels of neuroimmune peptides such as substance P and vasoactive intestinal peptide implicate neuroimmune interactions in disease severity [58].

The derived ratios and inflammatory indices did not differ significantly between our surgical and non-surgical groups, again at odds with prior work in which they carried prognostic value: elevated NLR and PLR have been associated with greater disease severity and intestinal ischemia, and low LCR and high CAR with necrosis and the need for surgery [22,35,36,38,40,45,49,59]. In the three-group analysis, NLR, MLR, PLR, and SII did reach statistical significance, but these differences arose between the spontaneously resolving and hydrostatically reduced groups rather than the surgical group, so the ratios did not identify the need for surgery. The HALP score is inversely related to disease severity, with lower scores signaling a higher likelihood of complications [36], and in the study by Liu et al. the systemic immune-inflammation index (SII), derived from platelets, neutrophils, and lymphocytes, was highly sensitive for predicting the need for surgery [60].

Children requiring surgery also showed significant acid-base and metabolic changes, chiefly lower serum potassium and higher glucose, in keeping with the literature linking electrolyte and metabolic derangements to more severe disease. In the multivariable model, lower potassium remained independently associated with the need for surgery (OR 0.52 per 1 − SD; 95% CI 0.29–0.91; *p* = 0.021). Hypokalemia has been independently associated with bowel ischemia and failed non-operative reduction, likely reflecting systemic and local ischemic processes that disturb electrolyte homeostasis [61], while the elevated glucose seen in our surgical group may represent a stress response accompanying severe inflammation and tissue hypoperfusion [62,63]. Other markers, hyponatremia, hypokalemia, and elevated lactic acid, have been reported as indicators of intestinal ischemia and necrosis [51,61,64,65], and serum albumin and TBA have shown predictive value for ischemic complications, underscoring the value of comprehensive biochemical assessment in risk stratification [38,66].

Children requiring surgery had significantly lower erythrocyte, hemoglobin, and hematocrit levels than those reduced spontaneously or hydrostatically, and multivariate analysis identified a low erythrocyte count as an independent predictor of the need for surgery (OR 0.58 per 1 − SD; 95% CI 0.34–0.99; *p* = 0.045). This accords with earlier work in which anemia-related parameters, particularly hemoglobin below 12.2 g/dL, were associated with failure of non-operative reduction in pediatric ileocolic intussusception [43,67]. Reduced erythrocyte and hemoglobin levels may reflect an underlying inflammatory or ischemic process that hampers reduction and necessitates surgery. Nonetheless, although these markers are accessible and inexpensive, prospective studies are needed to validate their accuracy and clarify the mechanisms linking anemia to treatment outcome.

Beyond routine indices, serum biomarkers such as alpha-GST and I-FABP show particular promise. Alpha-GST above 3.29 ng/mL is highly indicative of cases unlikely to resolve spontaneously, with excellent diagnostic accuracy [49], and elevated I-FABP correlates with bowel necrosis, a cut-off around 1538 ng/mL offering moderate predictive value for resection [68]. D-dimer and fibrinogen, above 0.24–1 mg/L and 1.26 g/L respectively, have been reported as independent predictors of ischemic and necrotic complications [23,53,66,69,70], and emerging markers such as serum transfer RNA fragments (tRFs) show promise for the non-invasive diagnosis of bowel necrosis [71].

Given the moderate predictive power of admission erythrocyte and platelet counts demonstrated in our study, their integration into clinical algorithms could enhance early risk stratification when combined with clinical signs and imaging findings. For example, patients presenting with low erythrocyte counts and high platelet levels, alongside ultrasound indicators of bowel edema, ischemia, or free fluid, may be identified as being at higher risk for requiring surgical intervention. Incorporating these hematological biomarkers into a composite scoring system or decision-support tool could facilitate the timely identification of children who may benefit from early surgical consultation, supporting rather than replacing clinical judgment. Prospective validation of such integrated algorithms could lead to more precise, individualized treatment pathways, reducing delays in definitive care and potentially improving clinical outcomes in pediatric intussusception.

The study has several limitations. The relatively small sample size, especially within subgroups, may limit the statistical power to detect subtle but clinically relevant differences and limit robust multivariate analysis. Because a large panel of biomarkers was screened, the univariable group comparisons are subject to multiple-comparison error; although we controlled the false discovery rate with the Benjamini–Hochberg procedure, the exploratory associations that survived correction, and, a fortiori, the six that did not (MCH, MCHC, absolute lymphocyte and eosinophil counts, the immature-granulocyte fraction, and SIRI), should be interpreted as hypothesis-generating and require confirmation in adequately powered, prospectively designed studies. The study’s reliance on biomarkers at the time of admission precludes the assessment of dynamic changes during disease progression, which may provide more accurate prognostic information. Importantly, the considerable heterogeneity in the interval between symptom onset and hospital admission may significantly influence the levels of inflammatory and hematologic markers, thereby confounding their association with treatment outcomes. Moreover, the modest ROC AUC values for erythrocyte and platelet counts suggest limited independent predictive accuracy, raising questions about their clinical utility when used in isolation. Serum potassium showed the highest individual discrimination (AUC 0.70; *p* < 0.001), exceeding the erythrocyte and platelet counts, although it likewise reflects only moderate accuracy and is best used within a multivariable model rather than in isolation. Consistent with this, the diagnostic likelihood ratios were modest, and the negative likelihood ratios (0.64–0.77) lay close to 1, so neither marker can reliably exclude the need for surgery on its own. These parameters should therefore be regarded as candidate variables for incorporation into multivariable or composite prediction models rather than as standalone, clinically actionable triage tests. In addition, the study does not account for potential confounding factors such as nutritional status or hydration level that may affect hematological and inflammatory markers independently of intussusception severity. The absence of external validation cohorts or prospective validation reduces the power of the results, highlighting the need for future studies to confirm the utility of these biomarkers in different settings. Because radiological interpretation was retrospective and each examination was generally read by a single radiologist, inter-observer reliability of the index test could not be assessed. The moderate agreement between the ultrasound-based and intraoperative anatomical classification observed here (κ = 0.59) is consistent with the published performance of ultrasound, which has excellent sensitivity and specificity for detecting intussusception but is recognized to be less precise in establishing the exact anatomical type and in identifying a pathological lead point. Together, these limitations highlight the need for larger, prospective, multicenter studies with serial measurements of biomarkers and comprehensive control of confounding factors to confirm and improve the predictive value of these biomarkers in the management of intussusception in children.

## 5. Conclusions

In this retrospective cohort of children with intussusception, lower erythrocyte counts, higher platelet counts, and lower serum potassium at admission independently predicted the need for surgery. Although their predictive performance was only moderate, these parameters are simple, inexpensive, and routinely available. However, their modest standalone discrimination means they are best used as candidate variables within multivariable or composite prediction models rather than as standalone triage markers. Prospective, multicenter studies, ideally incorporating dynamic biomarker monitoring and adjustment for confounders, are needed to confirm these findings and to build and validate such models for clinical use.

## Figures and Tables

**Table 1:** Demographic and clinical data of patients at admission between groups (*n* = 141).

Variable	Group 1 (*n* = 48)	Group 2 (*n* = 39)	Group 3 (*n* = 54)	*p*
Age (months)				
mean ± SD	61 ± 42	23 ± 12	36 ± 47	<0.001 * (G_1_/G_2_, G_1_/G_3_)
Sex male, *n* (%)				
	26 (54.2)	26 (66.7)	32 (59.3)	0.497 ^‡^
Comorbidity ^^^, *n* (%)				
Yes	2 (4.2)	4 (10.3)	5 (9.3)	0.526 ^†^
No	46 (95.8)	35 (89.7)	49 (90.7)	
Infection, *n* (%)				
Yes	9 (18.8)	12 (30.8)	11 (20.4)	0.360 ^‡^
No	39 (81.2)	27 (69.2)	43 (79.6)	
Antibiotic therapy, *n* (%)				
Yes	2 (4.2)	4 (10.3)	6 (11.1)	0.409 ^†^
No	46 (95.8)	35 (89.7)	48 (88.9)	
Season, *n* (%)				
Spring	8 (16.7)	9 (23.1)	18 (33.3)	0.458 ^‡^
Summer	10 (20.8)	8 (20.5)	10 (18.5)	
Autumn	18 (37.5)	12 (30.8)	11 (20.4)	
Winter	12 (25)	10 (25.6)	15 (27.8)	
Duration of abdominal pain, *n* (%)				
<24 h	23 (47.9)	19 (48.7)	16 (29.6)	0.101 ^‡^
24–48 h	10 (20.8)	11 (28.2)	12 (22.2)	
>48 h	15 (31.3)	9 (23.1)	26 (48.1)	
Intermittent pattern of pain, *n* (%)				
Yes	18 (37.5)	31 (79.5)	28 (51.9)	<0.001 ^‡^
No	30 (62.5)	8 (20.5)	26 (48.1)	
VAS	2.1 ± 0.9	3.2 ± 1.9	2.6 ± 1.4	0.075 ^#^
Nausea or vomiting, *n* (%)				
Yes	27 (56.2)	27 (69.2)	39 (72.2)	0.207 ^‡^
No	21 (43.8)	12 (30.8)	15 (27.8)	
Stool consistency, *n* (%)				
Normal	32 (66.7)	25 (64.1)	37 (68.5)	0.563 ^‡^
Diarrhea	10 (20.8)	12 (30.8)	14 (25.9)	
Hard	6 (12.5)	2 (5.1)	3 (5.6)	
Presence of bloody stools, *n* (%)				
Yes	1 (2.1)	3 (7.7)	21 (38.9)	<0.001 ^†^
No	47 (97.9)	36 (92.3)	33 (61.1)	
Body temperature, *n* (%)				
<38.0 °C	45 (93.8)	39 (100.0)	50 (92.6)	0.231 ^†^
≥38.0 °C	3 (6.2)	0 (0.0)	4 (7.4)	
Palpatory abdominal tenderness, *n* (%)				
Yes	22 (45.8)	8 (20.5)	23 (42.6)	0.033 ^‡^
No	26 (54.2)	31 (79.5)	31 (57.4)	

G1—Group 1 (spontaneous reduction), G2—Group 2 (hydrostatic reduction), G3—Group 3 (surgical treatment), VAS—Visual Analog Scale, * Kruskal–Wallis test, ^‡^ Chi-square test, ^†^ Fisher’s exact test, ^#^ ANOVA, ^^^ Comorbidity refers to any additional pre-existing or concurrent medical condition present at admission alongside the intussusception that was not an exclusion criterion for the study.

**Table 2:** Ultrasound characteristics upon admission between groups (*n* = 141).

Variable	Group 1 (*n* = 48)	Group 2 (*n* = 39)	Group 3 (*n* = 54)	*p*
Type of intussusception, *n* (%)				
Ileocolic	3 (6.2)	38 (97.4)	37 (68.5)	<0.001 ^‡^
Ileocecocolic	0 (0)	0 (0)	4 (7.4)	
Ileoileal	42 (87.5)	0 (0)	11 (20.3)	
Jejunojejunal	2 (4.2)	0 (0)	1 (1.9)	
Ileocolic + ileoileal	0 (0)	1 (2.6)	1 (1.9)	
Ileoileal + jejunojejunal	1 (2.1)	0 (0)	0 (0)	
Size (cm)	2.5 ± 1.2	5.4 ± 3.9	5.8 ± 4.3	0.003 * (G_1_/G_2_, G_1_/G_3_)
Edema of the intestinal wall, *n* (%)				
Yes	15 (31.3)	5 (12.8)	23 (42.6)	0.008 ^‡^
No	33 (68.7)	34 (87.2)	31 (57.4)	
Ultrasound signs of ischemia, *n* (%)				
Yes	0 (0)	0 (0)	7 (13)	0.003 ^†^
No	48 (100)	39 (100)	47 (87)	
Presence of free fluid in the abdomen, *n* (%)				
Yes	13 (27.1)	7 (17.9)	24 (44.4)	0.018 ^‡^
No	35 (72.9)	32 (82.1)	30 (55.6)	

G_1_—Group 1 (spontaneous reduction), G_2_—Group 2 (hydrostatic reduction), G_3_—Group 3 (surgical treatment), * Kruskal–Wallis test, ^‡^ Chi-square test, ^†^ Fisher’s exact test.

**Table 3:** Blood count variables of patients at admission between groups (*n* = 141).

Variable	Group 1 (*n* = 48)	Group 2 (*n* = 39)	Group 3 (*n* = 54)	*p*	q (BH)	η^2^ (95% CI)
Erythrocytes (×10^12^/L)	4.74 ± 0.41	4.57 ± 0.40	4.40 ± 0.53	0.001 ^#^ (G_1_/G_3_)	0.008	0.09 (0.02–0.21)
Hemoglobin (g/L)	124.77 ± 11.20	115.87 ± 10.96	114.25 ± 15.99	<0.001 ^#^ (G_1_/G_2_, G_1_/G_3_)	0.007	0.11 (0.05–0.21)
Hematocrit (L/L)	0.38 ± 0.03	0.36 ± 0.03	0.35 ± 0.04	<0.001 ^#^ (G_1_/G_2_, G_1_/G_3_)	0.007	0.10 (0.03–0.23)
MCV (fL)	80.71 ± 5.23	79.48 ± 2.95	80.64 ± 4.96	0.387 ^#^	0.457	0.01 (0.00–0.08)
MCH (pg)	26.38 ± 1.94	25.38 ± 1.40	25.96 ± 1.94	0.041 ^#^ (G_1_/G_2_)	0.098	0.05 (0.01–0.15)
MCHC (g/L)	326.85 ± 12.18	319.46 ± 12.27	321.78 ± 14.73	0.029 ^#^ (G_1_/G_2_)	0.076	0.05 (0.01–0.15)
RDW-CV (%)	13.77 ± 1.30	13.62 ± 1.14	13.61 ± 1.42	0.814 ^#^	0.832	0.00 (0.00–0.07)
Platelets (×10^9^/L)	313.29 ± 97.17	328.74 ± 76.91	389.92 ± 136.39	0.001 ^#^ (G_1_/G_3_, G_2_/G_3_)	0.008	0.09 (0.03–0.20)
MPV (fL)	9.16 ± 0.80	9.02 ± 0.71	9.10 ± 0.70	0.492 ^#^	0.553	0.01 (0.00–0.09)
Leukocytes (×10^9^/L)	12.12 ± 5.30	10.92 ± 3.84	12.29 ± 5.06	0.370 ^#^	0.449	0.01 (0.00–0.08)
Neutrophils						
(%)	67.70 ± 16.36	54.47 ± 17.06	60.56 ± 17.08	0.001 ^#^ (G_1_/G_2_)	0.008	0.09 (0.03–0.22)
(×10^9^/L)	8.58 ± 4.83	6.37 ± 3.65	7.62 ± 3.98	0.056 ^#^	0.130	0.04 (0.01–0.14)
Lymphocytes						
(%)	22.94 ± 14.18	35.51 ± 14.69	29.49 ± 15.08	<0.001 ^#^ (G_1_/G_2_)	0.007	0.11 (0.04–0.24)
(×10^9^/L)	2.48 ± 1.41	3.52 ± 1.23	3.51 ± 2.83	0.018 ^#^ (G_1_/G_2_, G_1_/G_3_)	0.060	0.06 (0.02–0.16)
Monocytes						
(%)	7.53 ± 2.92	8.61 ± 3.42	9.09 ± 4.40	0.113 ^#^	0.217	0.03 (0.00–0.12)
(×10^9^/L)	0.86 ± 0.40	0.89 ± 0.38	1.08 ± 0.64	0.071 ^#^	0.145	0.04 (0.00–0.14)
Eosinophils						
(%)	1.47 ± 1.88	1.03 ± 1.19	0.55 ± 0.96	0.008 ^#^ (G_1_/G_3_)	0.035	0.07 (0.02–0.17)
(×10^9^/L)	0.16 ± 0.22	0.10 ± 0.13	0.07 ± 0.12	0.021 ^#^ (G_1_/G_3_)	0.063	0.06 (0.01–0.15)
Basophils						
(%)	0.35 ± 0.16	0.38 ± 0.26	0.31 ± 0.17	0.269 ^#^	0.345	0.02 (0.00–0.10)
(×10^9^/L)	0.04 ± 0.02	0.04 ± 0.02	0.04 ± 0.03	0.720 ^#^	0.771	0.01 (0.00–0.07)
IG						
(%)	0.21 ± 0.14	0.26 ± 0.18	0.30 ± 0.15	0.023 ^#^ (G_1_/G_3_)	0.064	0.06 (0.01–0.16)
(×10^9^/L)	0.03 ± 0.02	0.03 ± 0.03	0.04 ± 0.03	0.206 ^#^	0.288	0.03 (0.00–0.12)
CRP (mg/L)	12.34 ± 25.46	5.99 ± 6.99	13.12 ± 20.71	0.363 ^#^	0.449	0.02 (0.01–0.12)

G_1_—Group 1 (spontaneous reduction), G_2_—Group 2 (hydrostatic reduction), G_3_—Group 3 (surgical treatment), MCV—mean corpuscular volume, MCH—mean corpuscular hemoglobin, MCHC—mean corpuscular hemoglobin concentration, RDW-CV—red cell distribution width—coefficient of variation, MPV—mean platelet volume, g—gram, L—liter, fL—femtoliter, pg—picogram, IG—immature granulocytes, CRP—C-reactive protein, mg—milligram, ^#^ ANOVA, q—Benjamini–Hochberg false-discovery-rate–adjusted *p*-value across the 46 exploratory three-group comparisons, η^2^—eta-squared effect size with 95% bootstrap confidence interval.

**Table 4:** Patient ratios and indexes at admission (*n* = 141).

Variable	Group 1 (*n* = 48)	Group 2 (*n* = 39)	Group 3 (*n* = 54)	*p*	q (BH)	η^2^ (95% CI)
NLR	5.11 ± 4.58	2.22 ± 2.18	3.12 ± 2.67	<0.001 ^#^ (G_1_/G_2_, G_1_/G_3_)	0.007	0.12 (0.04–0.23)
NMR	10.93 ± 6.13	8.19 ± 5.96	9.61 ± 8.84	0.213 ^#^	0.289	0.02 (0.00–0.12)
MLR	0.43 ± 0.25	0.27 ± 0.12	0.36 ± 0.20	0.001 ^#^ (G_1_/G_2_)	0.008	0.09 (0.04–0.19)
PLR	169.69 ± 111.11	105.62 ± 53.39	138.61 ± 54.93	0.014 ^#^ (G_1_/G_2_)	0.008	0.10 (0.03–0.20)
SII (×10^9^/L)	1547.95 ± 1373.28	735.49 ± 717.32	1105.48 ± 767.22	0.001 ^#^ (G_1_/G_2_)	0.008	0.10 (0.03–0.21)
SIRI (×10^9^/L)	4.02 ± 3.24	2.49 ± 2.25	2.92 ± 2.29	0.023 ^#^ (G_1_/G_2_)	0.064	0.06 (0.01–0.16)

G_1_—Group 1 (spontaneous reduction), G_2_—Group 2 (hydrostatic reduction), G_3_—Group 3 (surgical treatment), NLR—Neutrophil-to-Lymphocyte Ratio, NMR—Neutrophil-to-Monocyte Ratio, MLR—Monocyte-to-Lymphocyte Ratio, PLR—Platelet-to-Lymphocyte Ratio, SII—Systemic Immune-Inflammation Index, SIRI—Systemic Inflammatory Response Index, L—liter, ^#^ ANOVA, q—Benjamini–Hochberg false-discovery-rate–adjusted *p*-value across the 46 exploratory three-group comparisons, η^2^—eta-squared effect size with 95% bootstrap confidence interval.

**Table 5:** Acid-base status variables of patients at admission between groups (*n* = 141).

Variable	Group 1 (*n* = 48)	Group 2 (*n* = 39)	Group 3 (*n* = 54)	*p*	q (BH)	η^2^ (95% CI)
pH	7.40 ± 0.07	7.39 ± 0.04	7.39 ± 0.08	0.849 ^#^	0.849	0.00 (0.00–0.09)
pCO_2_ (kPa)	4.36 ± 0.71	4.12 ± 0.62	4.46 ± 0.86	0.194 ^#^	0.280	0.03 (0.00–0.13)
pO_2_ (kPa)	9.51 ± 2.46	10.13 ± 2.22	8.95 ± 2.95	0.195 ^#^	0.280	0.03 (0.00–0.15)
HCO_3_ (mmol/L)	21.00 ± 2.25	20.11 ± 1.98	20.76 ± 2.97	0.397 ^#^	0.457	0.02 (0.00–0.11)
Total CO_2_ (mmol/L)	20.83 ± 2.52	19.44 ± 2.81	20.73 ± 3.01	0.119 ^#^	0.220	0.04 (0.01–0.16)
BE (mmol/L)	−4.01 ± 2.78	−4.91 ± 2.03	−4.39 ± 3.62	0.540 ^#^	0.592	0.01 (0.00–0.10)
sO_2_ (%)	92.07 ± 9.04	93.13 ± 6.73	88.29 ± 12.07	0.118 ^#^	0.258	0.04 (0.01–0.15)
Potassium (mmol/L)	4.36 ± 0.47	4.55 ± 0.35	4.15 ± 0.42	<0.001 ^#^ (G_2_/G_3_)	0.007	0.14 (0.06–0.29)
Sodium (mmol/L)	138.72 ± 2.20	138.23 ± 2.49	137.34 ± 3.77	0.162 ^#^	0.258	0.04 (0.00–0.14)
Chlorides (mmol/L)	104.62 ± 2.54	105.08 ± 3.27	103.17 ± 4.08	0.064 ^#^	0.139	0.06 (0.01–0.18)
Calcium (mmol/L)	1.26 ± 0.04	1.26 ± 0.03	1.24 ± 0.07	0.190 ^#^	0.280	0.04 (0.00–0.16)
Glucose (mmol/L)	4.89 ± 0.93	4.71 ± 0.98	6.23 ± 1.94	<0.001 ^#^ (G_1_/G_3_, G_2_/G_3_)	0.002	0.20 (0.09–0.35)
Lactates (mmol/L)	1.63 ± 0.56	1.53 ± 0.34	1.80 ± 0.91	0.254 ^#^	0.335	0.03 (0.00–0.10)
Oxyhemoglobin (%)	91.20 ± 9.00	92.14 ± 6.59	87.43 ± 11.96	0.122 ^#^	0.258	0.04 (0.01–0.13)
Carboxyhemoglobin (%)	0.42 ± 0.26	0.39 ± 0.19	0.38 ± 0.25	0.778 ^#^	0.813	0.01 (0.00–0.10)
Methemoglobin (%)	0.51 ± 0.24	0.67 ± 0.21	0.64 ± 0.33	0.072 ^#^	0.145	0.06 (0.01–0.18)
Deoxyhemoglobin (%)	9.73 ± 13.32	6.80 ± 6.69	13.12 ± 15.97	0.162 ^#^	0.258	0.04 (0.01–0.13)

G_1_—Group 1 (spontaneous reduction), G_2_—Group 2 (hydrostatic reduction), G_3_—Group 3 (surgical treatment), pCO_2—_partial pressure of carbon dioxide, pO_2_—partial pressure of oxygen, HCO_3_—bicarbonate, BE—base excess, sO_2_—oxygen saturation, kPa—kilopascal, mmol—millimole, L—liter, ^#^ ANOVA, q—Benjamini–Hochberg false-discovery-rate–adjusted *p*-value across the 46 exploratory three-group comparisons, η^2^—eta-squared effect size with 95% bootstrap confidence interval.

**Table 6:** Multiple binary logistic regression analysis.

Variable	Odds Ratio	Standard Error	z	*p*-Value	Lower Bound (95%)	Upper Bound (95%)
Erythrocytes	0.58	0.271	−2.00	0.045	0.34	0.99
Platelets	1.97	0.312	2.18	0.029	1.07	3.64
Lymphocytes	0.91	0.306	−0.29	0.771	0.50	1.67
Potassium	0.52	0.286	−2.31	0.021	0.29	0.91

OR—odds ratio per one-standard-deviation (1 − SD) increase; z—ratio of the regression coefficient to its standard error; Lower/Upper Bound—95% confidence interval for the odds ratio.

## Data Availability

The data that support the findings of this study are available upon request from the corresponding author.

## Supplementary Materials

The following supporting information can be downloaded at https://www.mdpi.com/article/10.3390/diagnostics16152431/s1, Table S1: Restricted cohort (ileocolic and ileocecocolic intussusception, *n* = 82): demographic, clinical, ultrasound, and laboratory characteristics by treatment outcome; Table S2: Multivariable logistic regression and ROC analysis for the prediction of surgical treatment in the restricted cohort (ileocolic and ileocecocolic intussusception).

## Abbreviations

The following abbreviations are used in this manuscript:

ICD-10	International Classification of Diseases, 10th Revision
pH	Potential of Hydrogen
pCO_2_	Partial pressure of carbon dioxide
pO_2_	Partial pressure of oxygen
HCO_3_	Bicarbonate
BE	Base excess
sO_2_	Oxygen saturation
CRP	C-reactive protein
NLR	Neutrophil-to-Lymphocyte Ratio
NMR	Neutrophil-to-Monocyte Ratio
MLR	Monocyte-to-Lymphocyte Ratio
PLR	Platelet-to-Lymphocyte Ratio
SII	Systemic Immune-Inflammation Index
SIRI	Systemic Inflammatory Response Index
LOS	Length of stay
ANOVA	Analysis of Variance
SD	Standard Deviation
IQR	Interquartile Range
ROC	Receiver Operating Characteristic
AUC	Area Under the Curve
SE	Standard Error
PPV	Positive Predictive Value
NPV	Negative Predictive Value
Min	Minimum
Max	Maximum
IL	Interleukin
TNF-α	Tumor necrosis factor-α
MCP-1	Monocyte chemoattractant protein-1
LCR	Lymphocyte-to-C-reactive protein ratio
CAR	C-reactive protein/albumin ratio
TBA	Total bile acid
alpha-GST	Alpha-glutathione S-transferase
I-FABP	Intestinal fatty-acid-binding protein
