# Supplementary material for: Biomarker-Based Prediction of Treatment Outcomes in Pediatric Intussusception: A Retrospective Cohort Study
Source: Diagnostics (Basel). 2026 Aug 1;16(15):2431. doi: 10.3390/diagnostics16152431 (PMC13464450; doi:10.3390/diagnostics16152431)
Supplement: Supplementary file 1 [file diagnostics-16-02431-s001.zip › diagnostics-4373231-supplementary.pdf]

Supplementary Materials:

Table S1. Restricted cohort (ileocolic and ileocecocolic intussusception, *n* = 82): demographic, clinical, ultrasound, and laboratory characteristics by treatment outcome.

Variable	Non-operative (*n* = 41)	Surgical (*n* = 41)	*p*
Age (months)	22 [18–29]	14 [8–28]	0.018 §
Symptom duration (hours)	24 [7–48]	55 [20–96]	0.006 §
Male sex, *n* (%)	27/41 (65.9%)	23/41 (56.1%)	0.497 †
Bloody stool, *n* (%)	3/41 (7.3%)	17/41 (41.5%)	<0.001 †
US signs of ischemia, *n* (%)	0/41 (0.0%)	4/41 (9.8%)	0.116 †
Bowel-wall edema, *n* (%)	8/41 (19.5%)	17/40 (42.5%)	0.032 †
Free fluid, *n* (%)	9/41 (22.0%)	17/41 (41.5%)	0.096 †
Suspected lead point, *n* (%)	1/41 (2.4%)	4/41 (9.8%)	0.359 †
Erythrocytes ($\times 10^{12}$/L)	4.55 [4.32–4.82]	4.29 [3.85–4.63]	0.020 §
Hemoglobin (g/L)	116 [107–122]	111 [98–118]	0.048 §
Hematocrit (L/L)	0.36 [0.34–0.38]	0.35 [0.32–0.37]	0.017 §
MCV (fL)	79.9 [77.6–81.4]	80.7 [76.2–84.1]	0.438 §
MCH (pg)	25.5 [24.2–26.6]	25.7 [24.6–26.8]	0.546 §
MCHC (g/L)	320 [310–330]	320 [311–329]	0.818 §
RDW-CV (%)	13.4 [13.0–14.1]	13.6 [12.9–14.4]	0.684 §
Platelets ($\times 10^{9}$/L)	318 [281–359]	379 [288–460]	0.005 §
MPV (fL)	9.1 [8.5–9.5]	9.0 [8.8–9.6]	0.589 §
Leukocytes ($\times 10^{9}$/L)	10.9 [8.3–13.2]	11.6 [8.2–14.7]	0.331 §
Neutrophils (%)	58.9 [41.4–69.1]	59.7 [48.8–69.2]	0.584 §
Neutrophils ($\times 10^{9}$/L)	6.0 [4.4–8.2]	6.6 [4.7–9.3]	0.315 §
Lymphocytes (%)	31.5 [24.0–43.2]	30.2 [19.4–38.3]	0.359 §
Lymphocytes ($\times 10^{9}$/L)	3.3 [2.4–4.3]	2.7 [2.3–4.3]	0.552 §
Monocytes (%)	8.4 [6.4–10.7]	9.1 [5.8–13.3]	0.518 §
Monocytes ($\times 10^{9}$/L)	0.80 [0.66–1.17]	1.02 [0.61–1.55]	0.180 §
Eosinophils (%)	0.70 [0.20–1.60]	0.10 [0.00–0.80]	0.020 §
Eosinophils ($\times 10^{9}$/L)	0.05 [0.02–0.15]	0.01 [0.00–0.07]	0.016 §
Basophils (%)	0.30 [0.20–0.50]	0.30 [0.10–0.40]	0.234 §
Basophils ($\times 10^{9}$/L)	0.03 [0.02–0.05]	0.02 [0.02–0.04]	0.342 §
IG (%)	0.20 [0.20–0.30]	0.30 [0.30–0.40]	0.049 §
IG ($\times 10^{9}$/L)	0.02 [0.01–0.04]	0.03 [0.02–0.05]	0.066 §
CRP (mg/L)	4.1 [1.6–9.0]	6.0 [0.8–24.1]	0.472 §
NLR	1.9 [1.0–2.7]	2.0 [1.2–3.8]	0.456 §
NMR	6.7 [4.0–10.7]	6.0 [3.8–10.3]	0.708 §
MLR	0.24 [0.19–0.40]	0.29 [0.20–0.43]	0.343 §
PLR	95 [81–117]	122 [85–192]	0.055 §
SII	571 [335–986]	897 [477–1378]	0.071 §
SIRI	2.0 [1.3–2.9]	2.2 [0.9–3.7]	0.992 §
Potassium (mmol/L)	4.50 [4.28–4.80]	4.10 [3.80–4.33]	<0.001 §
Sodium (mmol/L)	138 [136–140]	138 [136–140]	0.633 §

Chlorides (mmol/L)	105 [103–108]	104 [102–106]	0.286 §
Calcium (mmol/L)	1.26 [1.24–1.27]	1.24 [1.18–1.28]	0.139 §
Glucose (mmol/L)	4.7 [4.2–5.2]	5.8 [4.9–7.2]	<0.001 §
Lactate (mmol/L)	1.48 [1.28–1.68]	1.46 [1.19–1.93]	0.795 §

US – ultrasound, MCV - mean corpuscular volume, MCH - mean corpuscular hemoglobin, MCHC - mean corpuscular hemoglobin concentration, RDW-CV - red cell distribution width - coefficient of variation, MPV - mean platelet volume, g - gram, L - liter, fL - femtoliter, pg - picogram, IG - immature granulocytes, CRP - C-reactive protein, mg – milligram, NLR - Neutrophil-to-Lymphocyte Ratio, NMR - Neutrophil-to-Monocyte Ratio, MLR - Monocyte-to-Lymphocyte Ratio, PLR - Platelet-to-Lymphocyte Ratio, SII - Systemic Immune-Inflammation Index, SIRI - Systemic Inflammatory Response Index, mmol – millimole, § Mann–Whitney U test; † Fisher's exact test. The non-operative group comprises patients with spontaneous resolution (*n* = 3) or successful hydrostatic reduction (*n* = 38).

Table S2. Multivariable logistic regression and ROC analysis for the prediction of surgical treatment in the restricted cohort (ileocolic and ileocecocolic intussusception).

Parameter	Adjusted OR (95% CI)	*p*	ROC performance
Erythrocytes	0.45 (0.25–0.80)	0.007	AUC 0.653 (0.531–0.774); cut-off ≤ 4.26 $\times 10^{12}$/L; sensitivity 50.0%, specificity 78.0%
Platelets	2.71 (1.34–5.49)	0.006	AUC 0.682 (0.564–0.800); cut-off ≥ 365 $\times 10^{9}$/L; sensitivity 60.5%, specificity 82.9%
Age	1.78 (0.92–3.45)	0.087	—
Symptom duration	1.26 (0.75–2.12)	0.382	—
